# The role of H4 flagella in *Escherichia coli* ST131 virulence

**DOI:** 10.1038/srep16149

**Published:** 2015-11-09

**Authors:** Asha Kakkanat, Makrina Totsika, Kolja Schaale, Benjamin L. Duell, Alvin W. Lo, Minh-Duy Phan, Danilo G. Moriel, Scott A. Beatson, Matthew J. Sweet, Glen C. Ulett, Mark A. Schembri

**Affiliations:** 1 School of Chemistry and Molecular Biosciences, University of Queensland, Brisbane, Queensland, Australia; 2 Australian Infectious Diseases Research Centre, University of Queensland, Brisbane, Queensland, Australia; 3 Institute of Health and Biomedical Innovation, School of Biomedical Sciences, Queensland University of Technology, Brisbane, Queensland, Australia; 4 Institute for Molecular Bioscience, University of Queensland, Brisbane, Queensland, Australia; 5 School of Medical Science, and Menzies Health Institute Queensland, Griffith University, Gold Coast, Australia

## Abstract

*Escherichia coli* sequence type 131 (ST131) is a globally dominant multidrug resistant clone associated with urinary tract and bloodstream infections. Most ST131 strains exhibit resistance to multiple antibiotics and cause infections associated with limited treatment options. The largest sub-clonal ST131 lineage is resistant to fluoroquinolones, contains the type 1 fimbriae *fimH*30 allele and expresses an H4 flagella antigen. Flagella are motility organelles that contribute to UPEC colonisation of the upper urinary tract. In this study, we examined the specific role of H4 flagella in ST131 motility and interaction with host epithelial and immune cells. We show that the majority of H4-positive ST131 strains are motile and are enriched for flagella expression during static pellicle growth. We also tested the role of H4 flagella in ST131 through the construction of specific mutants, over-expression strains and isogenic mutants that expressed alternative H1 and H7 flagellar subtypes. Overall, our results revealed that H4, H1 and H7 flagella possess conserved phenotypes with regards to motility, epithelial cell adhesion, invasion and uptake by macrophages. In contrast, H4 flagella trigger enhanced induction of the anti-inflammatory cytokine IL-10 compared to H1 and H7 flagella, a property that may contribute to ST131 fitness in the urinary tract.

Urinary tract infections (UTIs) are among the most frequently diagnosed human diseases, with an estimated 150 million cases annually across the globe^1^. Approximately 40–50% of women and 5% of men will develop a UTI at some stage in their lifetime^2^. UTI usually starts as a bladder infection (cystitis), but may also develop into acute kidney infection (pyelonephritis) or bloodstream infection (urosepsis). Recurrent UTI is also common, with approximately 30% of women with acute UTI reported to experience a recurrent episode within 3–4 months of the initial infection^3^. UTIs are increasingly associated with treatment failure due to the high frequency of infections caused by multidrug resistant (MDR) pathogens^4^,^5^.

Uropathogenic *Escherichia coli* (UPEC) is the major cause of all community and hospital acquired UTIs^6^,^7^. Over the last few decades, several pandemic clones of UPEC, including *E. coli* sequence type 131 (ST131), have emerged and spread worldwide^8^. *E. coli* ST131 is a globally disseminated MDR clone originally identified due to its close association with the spread of the *bla*_CTX-M-15_ extended spectrum β-lactamase (ESBL) gene^9^,^10^,^11^. *E. coli* ST131 strains containing the *bla*_CTX-M-15_ allele represent a highly prevalent and dominant fluoroquinolone resistant-FimH30 (*H*30) ST131 sublineage (referred to as clade C2^12^ or *H*30-Rx^13^). Like all UPEC, *E. coli* ST131 contain a mosaic composition of virulence genes. While genes encoding type 1 fimbriae are specifically required for the formation of intracellular bacterial communities (IBCs) and bladder colonization in the mouse UTI model^14^, the prevalence of other genes commonly associated with UPEC virulence is variable. This includes genes encoding for other fimbriae (e.g. P fimbriae, Afa adhesins), toxins (e.g. hemolysin, cytotoxic necrotizing factor-1) and iron-acquisition systems that utilise siderophores (e.g. enterobactin, salmochelin, aerobactin)^9^,^12^,^15^.

The majority of *E. coli* ST131 strains, including those from clade C2, possess a flagellar antigen that belongs to the H4 serogroup. Flagella are multicomponent, filamentous organelles comprised primarily of an extracellular major subunit flagellin protein termed FliC. In *E. coli*, the FliC protein is generally highly conserved in the N-and C-terminal regions, but hyper-variable within the central domain^16^. Fifty-six different FliC variants have been characterized based on this hyper-variable region and these variants define the *E. coli* H antigen diagnostic serotype marker^17^. Flagella also contribute to UPEC virulence, with a range of H-types frequently detected in clinical strains. This includes common H-types such as H1 (produced by the UPEC reference strain CFT073) and H7 (produced by the UPEC reference strain UTI89)^18^,^19^. In cystitis strain UTI89, H7 flagella enhance persistence in the urinary tract^20^ and are required for biofilm formation in the bladder^21^,^22^. H1 flagella contribute to ascension of pyelonephritis strain CFT073 from the bladder to the upper urinary tract^23^,^24^ and mediate invasion of the UPEC strain AL511 (H12) into renal collecting duct cells^25^.

As pathogen-associated molecular patterns (PAMPs), flagella also trigger activation of host innate immune cells such as macrophages and neutrophils. Cell surface recognition of flagellin is mediated by the pattern recognition receptor Toll-like receptor 5 (TLR5)^26^,^27^,^28^, whilst cytosolic flagellin is sensed by the NOD-like receptor family CARD-containing 4 (NLRC4) inflammasome^28^,^29^. Native flagella/flagellin from a variety of pathogens can stimulate the production of various cytokines, including TNF-α, IL -1β, IL-6 and IL-10^30^,^31^,^32^,^33^. We recently showed that some UPEC strains can survive within macrophages^34^ and that multiple genes involved in flagella biosynthesis are highly upregulated in the intramacrophage environment within hours post infection^35^. However, the ability of different flagellin (H) subtypes to enhance UPEC uptake by macrophages, or contribute to intramacrophage bacterial survival, has not been investigated.

The high prevalence of H4 flagellin in *E. coli* ST131 provides an opportunity to study the role of this flagella type in the fitness and virulence of a highly successful clonal lineage. Interestingly, many ST131 strains of H4 type also contain an ancestral flagellar locus termed flag-2^36^. The flag-2 locus has been identified in several Gram-negative species and resembles the lateral flagellar system previously described in *Aeromonas* and *Vibrio*^37^. Both the H4-encoding *fliC* gene and the flag-2 locus represent regions of the ST131 genome previously associated with recombination^12^,^38^. In this study, we have applied an innovative approach involving the genetic exchange of different *fliC* alleles to dissect the function of H4 flagella in *E. coli* ST131 and its interaction with bladder epithelial cells and macrophages. Our studies reveal an important role for multiple flagella subtypes in contributing to the intracellular niche of UPEC, as well as a dominant role for H4 flagella in driving IL-10 cytokine production from innate immune cells.

## Results

### EC958 contains two flagellar systems, however only the archetypical Flag-1 system is expressed and functional

To investigate the motility phenotype of *E. coli* ST131, we tested the ability of a large collection of H4 serotype strains for their ability to swim through soft agar. In total, 99.3% (135/136) of the strains examined were motile in this assay, with two strains exhibiting a significantly delayed swimming phenotype (Supplementary Fig. S1a). Analysis of a previously described draft genome sequence of the single non-motile strain revealed it contains a large Phi2 prophage insertion in the *fliT* flagella gene, and this was confirmed by PCR (Supplementary Fig. S1). Overall, this demonstrates that motility is a highly conserved phenotype among ST131 strains.

*E. coli* ST131 strains from the globally dominant clade C group possess genes with the potential to encode two flagellar systems, the archetypical Flag-1 system and the non-typical Flag-2 system. In order to determine the contribution of these two systems to ST131 motility, we generated a series of mutations in the prototype ST131 strain EC958. This included mutation of the Flag-1 major structural subunit *fliC* gene (EC958*fliC*), mutation of the Flag-2 major structural subunit *lafA* gene (EC958*lafA*) and a *fliC-lafA* double mutant (EC958*fliC lafA*). In agar-based swimming assays, only EC958*lafA* was motile, demonstrating the direct role of the archetypical Flag-1 flagellar system in EC958 motility (Supplementary Fig. S2a). Given the inability of the Flag-2 system to mediate motility, we also tested for expression of Flag-2 flagella by western blot analysis using an antibody specifically targeted against the LafA flagellin subunit. No expression of LafA was detected in whole cell lysates prepared from wild-type (WT) EC958 or EC958*fliC* following growth in LB broth or on LB soft agar (Supplementary Fig. S2b) at 28 °C and 37 °C. Taken together, these results indicate that the Flag-2 system of EC958 is not expressed under the conditions tested in this study, and that the motility phenotype of EC958 is mediated by Flag-1 flagella.

### Static culture of EC958 enhances type 1 fimbriae and flagella expression

**Figure 1:**
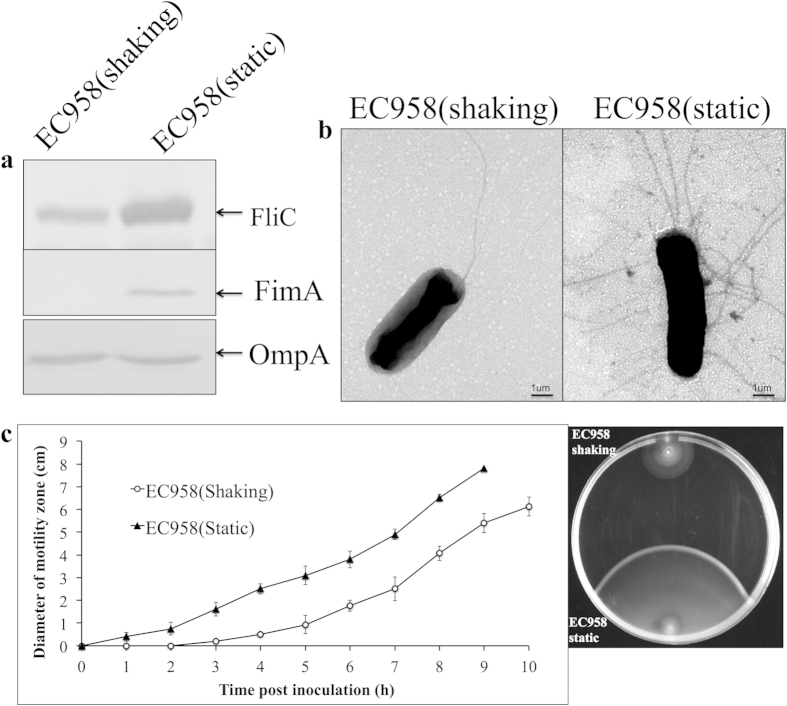
Static growth can enhance the flagellin expression and motility of EC958. (**a**) Western blot analysis of FliC (top panel) and FimA (bottom panel) performed using whole cell lysates prepared from EC958 cultured static or shaking in LB broth. OmpA was used as a loading control. (**b**) TEM image showing EC958 flagellation pattern under shaking and static growth conditions. (**c**) Graph demonstrating the rate of motility of EC958 in 0.25% LB agar following inoculation from static or shaking LB broth cultures (left). Comparative motility of the same EC958 cultures in 0.25% agar at 6 hpi (right). Data in a, b and c are representative experiments of three independent experiments.

The standard method for cultivation of UPEC to enrich for type 1 fimbriae expression involves several rounds of subculture in LB broth under static growth conditions. This enriches for type 1 fimbriated cells and results in the formation of a pellicle (or biofilm) at the air-liquid interface. To examine if this method of growth also enhanced flagella production by EC958, we compared its level of FliC and flagella expression following static versus shaking culture. Interestingly, we observed a significant increase in the expression of FliC flagellin following static growth of EC958 as determined by western blot analysis of whole cell lysates (Fig. 1a). These results were in line with the analysis of EC958 by transmission electron microscopy (TEM), which revealed a peritrichous arrangement of flagella on the majority of cells following growth under static conditions compared to the presence of a single (unilateral) flagella on the majority of cells following growth under shaking conditions (Fig. 1b). The increase in FliC during static growth was concomitant with an increase in the level of FimA (Fig. 1a).

In order to examine if the regulation of flagella expression in EC958 is coordinated with the production of type-1 fimbriae during static growth, we examined the transcript levels of *flhD* (encoding the flagella master regulator gene) and *fliC* in a wild-type and *fimD* mutant (EC958*fimD*) background. When EC958 was cultured under static growth conditions, the level of *flhD* (2.6-fold) and *fliC* (6.1-fold) transcription was significantly higher than observed during shaking growth (Supplementary Fig. S3). A similar increase in *flhD* (2.9-fold) and *fliC* (5.2-fold) transcription was also observed when EC958*fimD* was grown under static versus shaking conditions (Supplementary Fig. S3), indicating that flagellar gene transcription is increased in EC958 during static growth independent of type 1 fimbriae expression. In motility assays, cells from statically grown EC958 exhibited increased lateral swimming compared to cells grown under shaking conditions, further supporting the observation that static growth enriches for enhanced flagella production (Fig. 1c).

### Flagella enhance EC958 bladder cell adhesion and invasion

**Figure 2:**
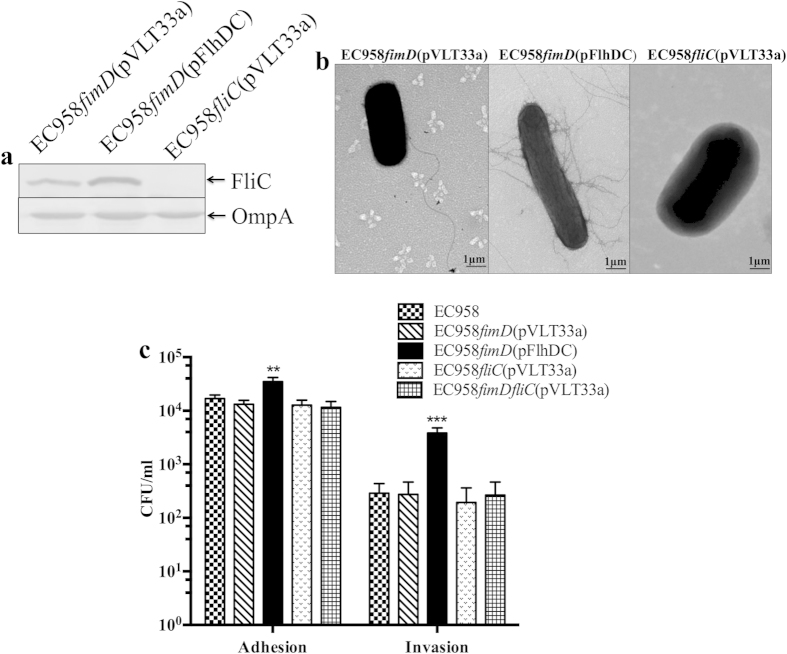
EC958 flagella expression leads to enhanced adhesion to and invasion of T24 bladder epithelial cells. (**a**) Western blot analysis of FliC performed using whole cell lysates prepared from EC958*fimD*(pVLT33a), EC958*fimD*(pFlhDC) and EC958*fliC*(pVLT33a), with OmpA used as loading control (**b**) TEM analysis demonstrating the pattern of flagella expression by EC958*fimD*(pVLT33a), EC958*fimD*(pFlhDC) and EC958*fliC*(pVLT33a). (**c**) Adhesion and invasion of EC958, EC958*fimD*(pVLT33a), EC958*fimD*(pFlhDC), EC958*fliC*(pVLT33a) and EC958*fimDfliC*(pVLT33a) to T24 bladder epithelial cells. Cell monolayers were infected at a multiplicity of infection (MOI) of 10; adherent and intracellular bacteria were enumerated by plating on LB agar. Shown is the mean adhesion and invasion in CFU/ml from three independent experiments  ± standard deviation (**p < 0.01; ***p < 0.001).

We previously showed that static culture of EC958 promotes adherence to and invasion of bladder epithelial cells^36^. Given the observation that static growth of EC958 induced both type 1 fimbriae and flagella expression, we next investigated if enhanced flagella expression contributed to these phenotypes. In order to avoid the effect of type 1 fimbriae, we used our EC958*fimD* mutant strain in combination with a plasmid containing the *flhDC* flagella master regulatory genes under the transcriptional control of the IPTG inducible ptac promoter (i.e. pFlhDC). Following IPTG induction, EC958*fimD*(pFlhDC) demonstrated enhanced expression of the FliC flagellin protein (Fig. 2a), produced flagella in a peritrichous arrangement (Fig. 2b) and was highly motile compared to the vector control strain EC958*fimD*(pVLT33a) (Supplementary Fig. S4). The role of flagella in adhesion and invasion was assessed using T24 human bladder epithelial cell monolayers. After induction with IPTG to induce flagellation, EC958*fimD*(pFlhDC) adhered to and invaded T24 bladder epithelial cells in significantly higher numbers than control strains EC958, EC958*fliC*(pVLT33a), EC958*fimD*(pVLT33a) and EC958*fimDfliC*(pVLT33a) (P < 0.01, P < 0.001 Fig. 2c). In these assays, EC958, EC958*fliC*(pVLT33a), EC958*fimD*(pVLT33a) and EC958*fimDfliC*(pVLT33a) adhered to T24 bladder epithelial cells at similar levels when cultured under shaking conditions (i.e. conditions that do not induce type 1 fimbriae expression). These data demonstrate that EC958 flagella contribute to adherence to and invasion of T24 bladder epithelial cells.

### Flagella contribute to EC958 uptake by macrophages

**Figure 3:**
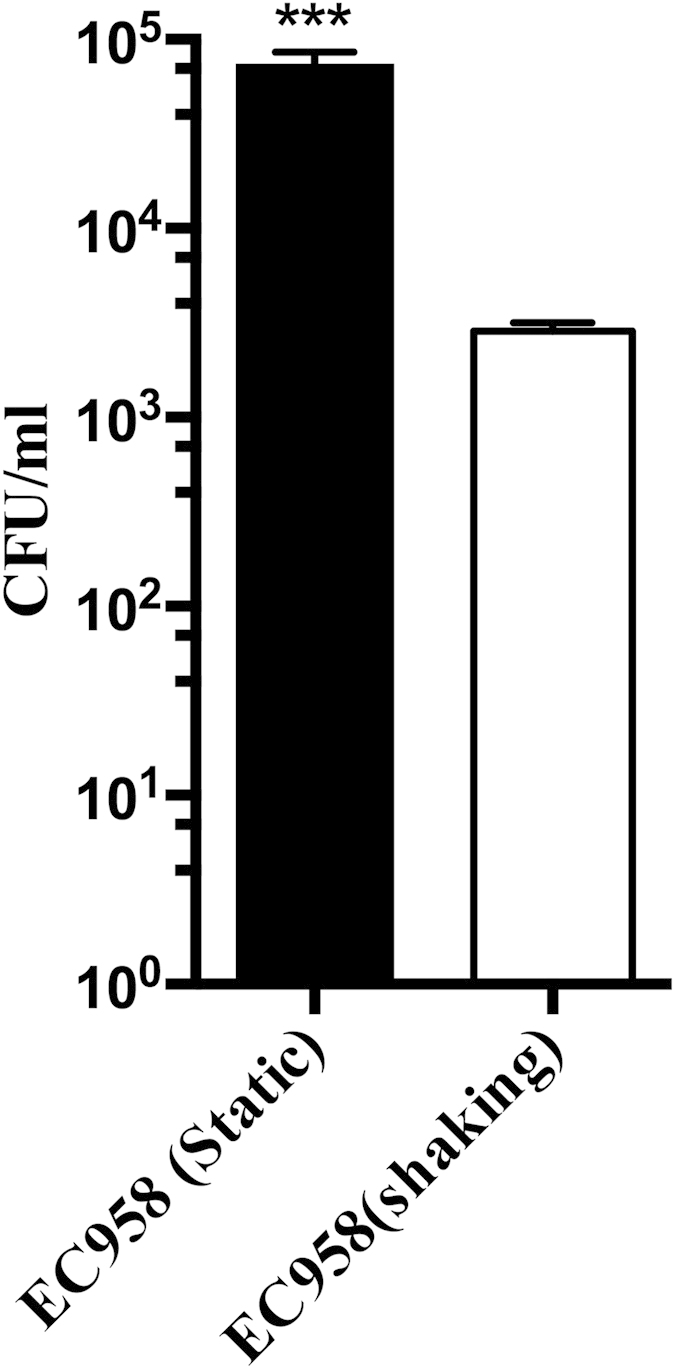
Static or shaking growth conditions can influence the uptake of EC958 by BMM. Uptake of EC958 by BMM (1 hpi). BMM were infected with EC958 at an MOI = 10 following static or shaking growth in LB broth. Shown is the mean uptake in CFU/ml from three independent experiments ± standard deviation (***p < 0.001).

**Figure 4:**
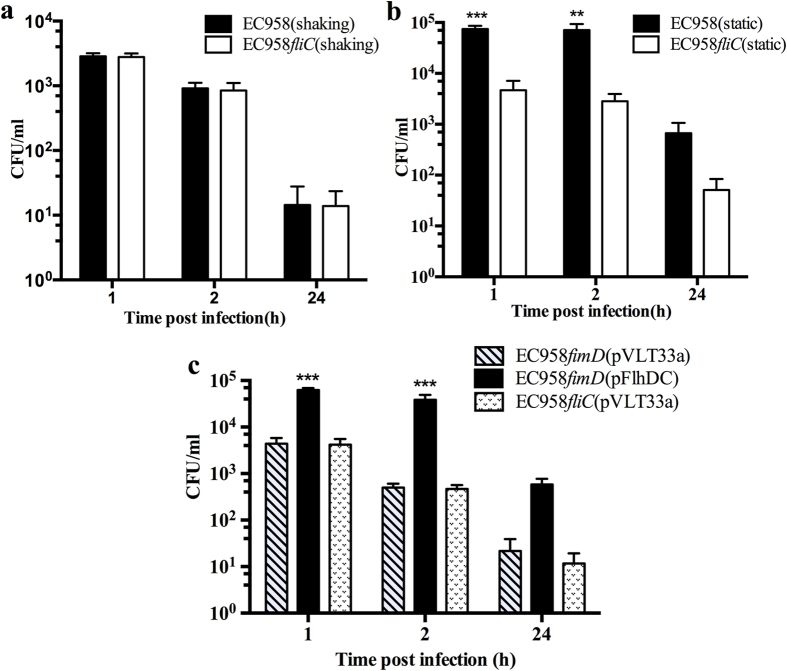
Comparative intramacrophage uptake and survival of EC958 and EC958*fliC* grown under (**a**) shaking and (**b**) static conditions. (**c**) Comparative intramacrophage uptake and survival of EC958*fimD*(pVLT33a), EC958*fimD*(pFlhDC) and EC958*fliC*(pVLT33a) grown under shaking conditions with IPTG induction. Triplicate monolayers of BMM were infected at a MOI of 10. Intracellular bacterial loads were determined at 1, 2 and 24 hpi. Shown is the mean survival in CFU/ml from three independent experiments ± standard deviation (***p < 0.001; **p < 0.01).

Some UPEC strains, such as the well-characterised recurrent cystitis isolate UTI89, can survive within mouse macrophages^34^. We hypothesized this would also be the case with EC958 and demonstrated that cells from a statically grown culture (enriched for type 1 fimbriae and flagella) were taken up in significantly higher numbers by BMM than cells of EC958 grown as a shaking culture (Fig. 3). In order to ascertain the contribution of flagella to EC958 uptake and survival in macrophages, we also infected BMM with our EC958*fliC* mutant cultured under shaking and static conditions. Following shaking growth, EC958*fliC* cells were taken up and survived in BMM in similar numbers to WT EC958 grown under the same conditions (Fig. 4a). In contrast, however, statically grown EC958*fliC* cells were taken up by BMM in significantly reduced numbers compared to WT EC958 grown under the same conditions (Fig. 4b). Relative to uptake, the intracellular load of statically grown EC958 was significantly higher than EC958*fliC* at 2 hours post infection (hpi), however no significant difference in bacterial load was observed at 24 hours post infection (Supplementary Fig. S5a). Thus, the enrichment of flagella expression during static growth of EC958 enhances its uptake by macrophages and early intramacrophage survival.

We next examined the contribution of flagella to UPEC uptake and survival in BMM in the absence of type 1 fimbriae using EC958*fimD*(pFlhDC) and EC958*fimD*(pVLT33a). The strains were cultured under shaking conditions, with flagella expression in EC958*fimD*(pFlhDC) induced by the addition of IPTG. Mouse BMM were infected with EC958*fimD*(pFlhDC) or EC958*fimD*(pVLT33a) at an MOI of 10. At 1 hpi, EC958*fimD*(pFlhDC) was recovered in ~10-fold higher numbers than EC958*fimD*(pVLT33a), demonstrating a direct contribution of flagella to uptake by BMM (Fig. 4c). EC958*fimD*(pFlhDC) intracellular loads were also higher at 2 hpi, with 62% of internalized EC958*fimD*(pFlhDC) recovered compared to 12% of internalized EC958*fimD*(pVLT33a) (Fig. 4c, Supplementary Fig. S5b). At 24 hpi, the intramacrophage loads of both EC958*fimD*(pFlhDC) and EC958*fimD*(pVLT33a) were both low (Fig. 4c), with less than 1% of EC958*fimD*(pFlhDC) and EC958*fimD*(pVLT33a) recovered relative to uptake (Supplementary Fig. S5b). As a control for all experiments, BMM were also infected with EC958*fliC*(pVLT33a); in all experiments the uptake and survival of EC958*fliC* was essentially the same as observed for EC958*fimD*(pVLT33a). Taken together, the data are consistent with the contribution of flagella to uptake and early survival of EC958 in BMM.

### Generation of a set of *fliC* isogenic EC958 strains

H1 and H7 represent two of the most common flagella serotypes of UPEC^17^. In contrast, most *E. coli* ST131 strains, including those from the dominant fluoroquinolone resistant-FimH30 clade C group, possess H4 serotype flagella^12^,^36^. Comparative sequence analysis of multiple FliC alleles demonstrated that the H4 FliC allele exhibits significant sequence variation compared to the H1 and H7 FliC alleles (Supplementary Fig. S6). In order to determine if the EC958 bladder epithelial cell invasion and macrophage uptake/survival phenotypes described above were specifically attributed to H4 flagella, we utilized a markerless gene replacement method to exchange the *fliC* allele in EC958 (H4) with the corresponding allele from CFT073(H1) or UTI89(H7), and combined this with a motility based positive-selection screen to identify the resultant variants (Supplementary Fig. S7). This positive selection method proved highly efficient, and resulted in the production of a set of isogenic EC958 strains that differed only in their *fliC* allele. The strains were referred to as EC958^H1^, EC958^H7^ and EC958^H4^ (control strain identical to EC958 parent).

### Isogenic EC958 strains differing in *fliC* alleles exhibit similar phenotypes

**Figure 5:**
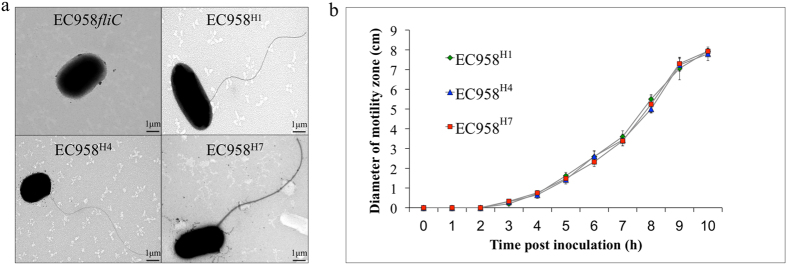
(**a**) TEM image demonstrating the production of flagella by EC958^H1^, EC958^H7^ and EC958^H4^, in comparison to the EC958*fliC* control strain. (**b**) Graph demonstrating the rate of motility of EC958^H1^, EC958^H7^, EC958^H4^ and EC958*fliC* in 0.25% LB agar. Shown is the mean diameter of the motility zone from three independent experiments ± standard deviation.

We compared EC958^H1^, EC958^H7^ and EC958^H4^ for their flagella morphology, motility, and interaction with epithelial cells and macrophages. All three EC958 derivative strains, but not the *fliC* mutant strain EC958*fliC*, expressed flagella observable by negative staining under transmission electron microscope (TEM) (Fig. 5a). Similarly, no significant difference was observed in the motility rate between these isogenic strains (Fig. 5b). The three strains also adhered to and invaded T24 bladder epithelial cells in equivalent numbers (Supplementary Fig. S8). The uptake and intracellular survival of these isogenic strains in mouse BMM and human macrophages (HMDM, THP-1 cells and U937 cells) was also examined, and no significant differences were observed (Supplementary Fig. S9). To test for differences associated with increased flagella expression, the strains were transformed with plasmid pFlhDC and tested for uptake and intracellular survival in macrophages. Similar to the observations above, flagellation enhanced bacterial uptake and the number of intracellular bacteria recovered from these macrophages. However, no significant differences were observed between EC958^H1^, EC958^H7^ and EC958^H4^ (Supplementary Fig. S9), demonstrating that variations in FliC do not affect these phenotypes.

### H4 flagellin enhances IL-10 production

**Figure 6:**
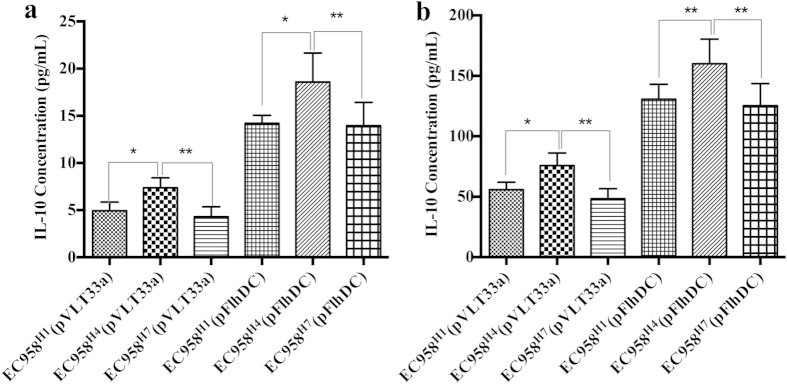
H4 flagella induce enhanced IL-10 secretion. (**a**) U937-derived monocytes and (**b**) co-cultures of 5367 uroepithelial cells and U937 monocytes were infected with EC958^H1^ (+/−pFlhDC), EC958^H7^ (+/−pFlhDC) or EC958^H4^ (+/−pFlhDC) for 5 hours, supernatant was recovered and IL-10 was measured by ELISA. Shown is the mean IL-10 concentration (pg/ml) from three independent experiments ± standard deviation (*p < 0.05; **p < 0.01; ***p < 0.001).

We have previously shown that co-cultures of bladder uroepithelial cells and monocytes produce IL-10 when challenged with UPEC^33^,^39^. In order to determine if flagella contribute to this response, we compared EC958^H1^, EC958^H7^ and EC958^H4^ for their ability to induce IL-10 secretion from U937 monocytes (Fig. 6a) and uroepithelial cell-U937 monocyte co-cultures (Fig. 6b). In both of these experimental settings, EC958^H4^ induced significantly higher IL-10 secretion compared to EC958^H1^ and EC958^H7^. Consistent with our previous results, a two-fold increase in IL-10 secretion was also observed when EC958^H1^, EC958^H7^ and EC958^H4^ were induced for flagella expression by transformation with plasmid pFlhDC. Taken together, these data show that H1, H4 and H7 flagella can induce IL-10 secretion, with H4 flagella the most potent flagellin type able to induce this cytokine.

## Discussion

The rapid emergence and global dissemination of *E. coli* ST131 has been associated with multiple factors, including increased antibiotic resistance, enhanced metabolic fitness, increased capacity to survive in the intestinal and/or urinary tract, and increased virulence^14^,^15^,^40^,^41^,^42^,^43^,^44^. Most ST131 strains produce H4 type flagella, a serotype also detected among strains of the recently emerged shiga toxin-producing *E. coli* pathotype that caused a large outbreak of acute gastroenteritis and haemolytic uraemic syndrome in Germany^45^. Here, we have shown that H4 flagella of the ST131 reference strain EC958 contribute to adherence to, and invasion of, bladder epithelial cells, and enhance bacterial uptake by macrophages. We also employed an innovative genetic exchange system to generate a set of isogenic mutants that express different flagellin subtypes and compared their phenotypic properties as well as their ability to stimulate the production of the immunoregulatory cytokine IL-10.

We screened a large collection of ST131 strains and demonstrated that the vast majority are motile. Our previous genomic analyses revealed that in addition to the archetypical flag-1 flagella system, many ST131 strains also contain a second flag-2 locus^12^,^36^. Flag-2 flagella were first described in the enteroaggregative *E. coli* strain O42 and resemble the lateral flagella systems of *Aeromonas* and *Vibrio* species^37^. The flag-2 gene clusters from EC958 and O42 share ~94% nucleotide sequence conservation. The major differences include an intact *lfgC* gene (putative chaperone) in EC958 compared to O42, and the absence of several genes in EC958, including *lafWZ* (putative toxin/antitoxin system)^37^, *maf-5* (motility/modification accessory factor required for lateral flagella function)^46^,^47^ and *motY* (stator component of flagella motor)^37^,^48^,^49^. We were unable to demonstrate the expression of Flag-2 flagella in EC958 under the experimental conditions employed in this study using both using motility and western blot assays. Thus, the primary mode of motility in ST131 appears to be mediated by the archetypical Flag-1 flagella system.

Static growth in liquid medium represents a standard method for the enrichment of type 1 fimbriated *E. coli* cells and leads to the formation of a pellicle at the air-liquid interface^50^. We have previously shown that EC958 contains an insertion in the *fimB* recombinase gene, and that this is associated with undetectable type 1 fimbriae expression during shaking growth, as well as a requirement for multiple successive rounds of static growth to enrich for type 1 fimbriae expression^36^. We compared the expression of the FliC major flagellin by western blot analysis following shaking and static growth of EC958 and observed a very strong increase in FliC production during static growth. Consistent with this result, static growth of EC958 also led to enhanced motility in soft agar and an enrichment for cells possessing a peritrichous pattern of flagella expression. Static growth of *Pseudomonas aeruginosa* has also been shown to enhance flagella production and this was linked to bacterial taxis towards higher oxygen concentrations at the air-liquid interface^51^. Interestingly, our results for EC958 differ to those recently reported for the UPEC reference strain UTI89, which requires the expression of functional flagella for confluent pellicle formation^22^,^52^. The different phenotypes associated with flagella expression during pellicle growth by EC958 and UTI89 may be due to a combination of factors, including differential type 1 fimbriae regulation between both strains, as well as differences in experimental design such as growth medium (LB broth for EC958 versus yeast extract casamino acids medium for UTI89) and the mode of pellicle enrichment (3 × 48 hours at 37 °C for EC958 versus 1x72 hours at 30 °C for UTI89)^36^,^52^.

Flagella contribute to multiple aspects of bacterial virulence, including motility, adhesion, biofilm formation and immune modulation^21^,^53^,^54^. In this work, several lines of evidence demonstrated that H4 flagella interact with both bladder epithelial cells and macrophages, which likely influences EC958 pathogenesis. First, the induction of flagella expression in an EC958*fimD* mutant resulted in increased adherence to and invasion of T24 bladder epithelial cells. Second, an EC958*fliC* mutant exhibited significantly reduced uptake by BMM compared to WT EC958. Finally, increased flagella expression significantly enhanced EC958 uptake by BMM independent of type 1 fimbriae expression. We recently reported that flagella genes are strongly upregulated at 2 hours post-infection within BMM, with a subsequent gradual decrease over time^35^. Our current results support the notion that the high level of flagella gene transcription by EC958 in the intramacrophage environment could be due to the selective uptake of flagellated UPEC. In mice, IBC formation by UTI89 does not require flagella^20^. However the same authors did identify a role for flagella in longer-term persistence using a competitive mixed infection assay. It would be interesting to examine the contribution of H4 flagella compared to other flagellar types during long-term bladder colonisation.

The FliC flagellin major subunit protein exhibits extensive sequence variation, a property that forms the basis of *E. coli* H-typing schemes. FliC sequence variation can also influence the interaction of flagella with host epithelial cells, and this has been demonstrated in the context of different *E. coli* pathotypes that express different H antigens. Some examples include H2 and H6 flagella from enteropathogenic *E. coli* (EPEC) and enterohemorrhagic *E. coli* (EHEC) that mediate adherence to intestinal epithelial cells^55^, and H6 and H7 flagella of EPEC and EHEC that mediate adherence to porcine gastric mucins and bovine primary intestinal epithelial cells^56^,^57^. In addition, H7 flagella mediate invasion of neonatal meningitis *E. coli* into human brain microvascular endothelial cells^58^ and the adherence of EHEC to bovine terminal rectal primary epithelial cells^57^. In order to compare the function of H4 flagella with other common flagella types associated with UPEC (namely H1 and H7), we devised a genetic system that enabled the markerless exchange of *fliC* alleles in EC958 in combination with the subsequent positive selection of motile mutants. This resulted in the generation of a set of EC958 isogenic strains that expressed H4 (WT), H1 and H7 flagella. In our experiments, there was no significant difference in H4-, H1-, and H7-mediated EC958 motility (assessed by swimming in soft agar), adherence to T24 bladder epithelial cells, invasion of T24 epithelial cells, and uptake and survival in murine and human macrophages. In addition, the induction of flagella expression by EC958^H1^, EC958^H7^ and EC958^H4^ enhanced all of these phenotypes, demonstrating that the interaction of H4, H1 and H7 flagellin with epithelial cells and macrophages occurs in a dose-dependent manner. Interestingly, a similar method for the genetic exchange of flagellin subunits was recently reported in *Salmonella* Typhi, and revealed that sequence differences in the three major *S*. Typhi flagellin antigens influence immunological responses to infection and impact on host cell invasion^59^. As this study only examined three common H types, the full extent of functional diversity among *E. coli* flagellins remains to be determined.

We hypothesized that there may be differences in the TLR5-mediated responses triggered in innate immune cells following exposure to H4 flagellin compared to H1 and H7 flagellin. Several recent studies have defined a role for the anti-inflammatory cytokine IL-10 in UPEC-mediated UTI^33^,^39^,^60^, and thus we investigated the capacity of EC958^H1^, EC958^H7^ and EC958^H4^ to induce IL-10 secretion in a co-culture model to take advantage of the interactions between different cell types in mixed infection assays^33^. Our analysis revealed that flagella comprised of H1, H4 and H7 flagellins induce IL-10 secretion. Most interestingly, however, flagella comprised of H4 flagellin induced a significantly stronger IL-10 response, an effect that may result from enhanced TLR5-dependent signalling. In mice, IL-10 is produced in the bladder by monocytes/macrophages^39^ and mast cells during the early stages of acute UTI. IL-10 acts by down-regulating pro-inflammatory responses and its ability to suppress humoral and cell-mediated responses may contribute to bacterial persistence^60^. It has also been observed that flagella are poorly expressed during chronic UTI in mice^61^. Thus, it is possible that H4 flagellin contributes to ST131 fitness by invoking an anti-inflammatory response during the early stages of UTI. Several recent studies have also demonstrated that some ST131 strains produce H5 flagella^62^,^63^,^64^, and we recently showed that these strains cluster into a less dominant divergent group (referred to as clade A)^12^. It would thus be interesting to characterise the properties of H5 flagella in ST131.

In conclusion, we have shown that H4 flagella contribute to several phenotypes associated with ST131 pathogenesis, including adherence and invasion (with respect to epithelial cells), uptake and intracellular survival (with respect to macrophages) and motility. While these phenotypes are not unique to H4 flagella, the finding that H4 flagella induce a strong IL-10 response suggests the flagella subtype may contribute to the overall fitness of the highly successful and globally dominant ST131 clone.

## Experimental procedures

### Bacterial strains and growth conditions

The strains and plasmids used in the study are listed in Table 1. The 136 ST131 strains tested for motility were part of our laboratory collection. All strains were cultured at 37 °C on solid or in liquid lysogeny broth (LB) medium, supplemented with the appropriate antibiotics (chloramphenicol 30 μg/ml or gentamicin 20 μg/ml), unless indicated otherwise. Strains containing the temperature sensitive plasmid pCP20 were cultured at 28 °C. Pellicle formation was induced by static growth in LB broth. Briefly, cells from a single colony were cultured for 48 hours, and then a volume of 10 μl was removed from the air-liquid interface and subcultured further two times under the same conditions.

### DNA manipulations and genetic techniques

Oligonucleotides used in this study were sourced from Integrated DNA Technologies (Singapore). All polymerase chain reactions were performed using One Taq DNA Polymerase (New England Biolabs) under standard conditions except for reactions requiring high fidelity, in which case KAPA HiFi DNA Polymerase (Biosystems) was used. DNA sequencing was performed using the Big Dye v3.1 kit (Applied Biosystems) at the Australian Equine Genetics Research Centre, University of Queensland. Isogenic mutations were constructed using λ-Red-mediated homologous recombination with some modifications^34^,^65^. The following primers were used for amplification of 500 bp upstream and downstream regions of *fliC* and *lafA*, respectively, from EC958: 3343 (5′-aagtaacccaatgccgcc), 3345 (5′-ggaccatggctaattcccatgacaaattccgctcctccc), 3346 (5′-gaagcagctccagcctacacctgcgccagagaaatacc), 3344 (5′-ccccaagcgttgaaatac) 3337 (5′-tttggcatcagtacattgag) 3339 (5′-ggaccatggctaattcccatgcatcgcggtagcatcatt), 3340 (5′-gaacagctccagcctacactggataccgattacgcgac), 3338 (5′-cattgatgactccggataac). Primers 3746 (5′-tcctccttagttcctattcc) and 3747 (5′-gtcttgagcgattgtgtagg) were used to generate the chloramphenicol resistance gene fragment from vector pKD3. The three fragments were fused together by PCR and this DNA fusion product was electroporated into EC958 harboring plasmid pKOBEG-Gent, and chloramphenicol resistant mutants were selected and confirmed by PCR using primers 3347 (5′-tcgtcgcgcataccaacc) and 3348 (5′-gatatcggcgtcagggtag), or 3341 (5′-tgtttcctgatagtgctttg) and 3342 (5′**-**cttctcctgcaaattcgtc). In order to generate a *fliC-lafA* double mutant, plasmid pCP20, expressing the FLP recombinase^66^, was transformed into a EC958*fliC* deletion mutant to remove the antibiotic resistance cassette prior to deletion of the *lafA* gene.

### Construction of isogenic *fliC*-allele swapped strains

A 3-way PCR procedure was employed to generate an amplification product that contained the *fliC* gene from CFT073 (*fliC*^H1^), UTI89 (*fliC*^H7^) or EC958 (*fliC*^H4^; control) flanked on both sides by ~500 bp of DNA sequence upstream and downstream of the EC958 *fliC* gene. The primers used to amplify the *fliC*^H1^ and *fliC*^H7^ from genomic DNA of CFT073 and UTI89 were 4967 (5′-aattccccttgtaggcctg), 4968 (5′-agggttgacggcgattgag) and 4969 (5′-ataagcacagcgcaccag), 4970 (5′-agggttgacggcgattgag), respectively. For amplification of DNA 500 bp upstream and downstream of the EC958 *fliC* gene, primers 4924 (5′-ctcagtttccgtggcgtt) and 4959 (5′-gttctgtctctgctgcagggttaatggccttaacctgcctgac) (upstream) or 4925 (5′-tggctaacgctaatggtg) and 4960 (5′-gctgttggtattaatgacttgtgccataattcattttcctgttttcaagtc) (downstream) were used. The PCR product was transformed into EC958*fliC* and integrated into the chromosome by λ-Red-mediated homologous recombination. In order to select for *fliC*-positive recombinants, 10 μl of the transformation mix was spotted onto 0.25% LB-agar, and motile swimming cells were recovered from the periphery of the plate after 18 h incubation. The allelic replacement mutants were confirmed by PCR screening and sequencing using the primers 4926 (5′-ctgttattggtgtcgagca) and 4927 (5′-tggcggctttactgtctt).

### qRT-PCR

Exponentially growing cells (OD_600nm_ = 0.6) were stabilized with two-volumes of RNAprotect Bacteria Reagent (Qiagen) prior to RNA extraction using the RNeasy Mini Kit (Qiagen) followed by on-column DNase digestion. Purified RNA samples were further treated with DNase I (Ambion) to ensure the complete removal of contaminating DNA, and re-purified using the RNeasy Mini Kit (Qiagen) RNA cleanup protocol. First-strand cDNA synthesis was performed using SuperScript® III First-Strand Synthesis System (Invitrogen) as per manufacturer’s recommendation. Real-time PCR was performed using SYBR® Green PCR Master Mix (Applied Biosystems) on the ViiA^TM^ 7 Real-Time PCR System (Applied Biosystems) using the following primers: *flhD*, primers 5613 (5′-acttgcacagcgtctgattg) and 5614 (5′-agcttaaccatttgcggaag); *fliC*, primers 5683 (5′-caccaacctgaacaacacca) and 5684 (5′-gcacggcgaatatccagttg). Transcript levels of each gene were normalized to *gapA* as the endogenous gene control (primers 820, 5′-ggtgcgaagaaagtggttatgac and 821, 5′-ggccagcatatttgtcgaagttag). Gene expression levels were determined using the 2^-ΔΔCT^ method with relative fold-difference expressed against EC958 (shaking).

### Mammalian cell culture and reagents

Murine bone marrow-derived macrophages (BMM) were generated by *in vitro* differentiation of bone marrow cells harvested from C57BL/6 mice in the presence of 50 ng/ml recombinant human CSF-1 (Peprotech), as described previously^34^,^67^. Human monocyte-derived macrophages (HMDM) were differentiated from CD14^+^ cells, as previously described^68^. Human monocytes were isolated from buffy coats of healthy donors (kindly provided by the Australian Red Cross) by positive selection for CD14 using MACS technology (Miltenyi Biotec, Bergisch Gladbach, Germany) as previously described^68^. THP-1 and U937 monocytes were purchased from American Type Culture Collection (ATCC) (Manassas, VA, USA) and were differentiated for 48 h with 50 ng/ml phorbol-12-myristate-13-acetate (PMA) (Sigma-Aldrich), as previously described^69^. BMM, HMDM, THP-1 and U937 cells were maintained in RPMI medium containing 10% heat-inactivated fetal bovine serum (FBS), 1% GLX solution (Life technologies), 20 U/ml penicillin and 20 μg/ml streptomycin (Invitrogen). T24 and 5637 bladder epithelial cells were maintained in modified McCoy’s 5A (Life technologies) or RPMI-1640 (Life technologies) respectively, supplemented with 10% heat inactivated FBS.

### Epithelial cell adhesion and invasion assay

T24 bladder epithelial cell adhesion and invasion assays were performed as previously described^70^. Briefly, confluent monolayers of T24 cells were infected at a multiplicity of infection (MOI) of 10, centrifuged at 180 × *g* for 2 minutes and then incubated at 37 °C, 5% CO_2_ for 1 hour. Non-adherent bacteria were removed by 5 washes with PBS. T24 cell lysates were serially diluted and plated onto LB agar plates to enumerate adherent bacteria. Enumeration of intracellular bacteria was performed in a similar manner following exclusion of extracellular bacteria by gentamicin treatment (200 mg/ml) for 1 hour.

### Macrophage infection assay

*In vitro* macrophage infection assays were carried out as previously described^34^. Briefly, following overnight adherence in antibiotic-free medium, cells were infected with bacteria at an MOI of 10 and centrifuged at 180 × *g* for 2 minutes. Infected cells were incubated at 37 °C, 5% CO_2_ for 1 hour. Extracellular bacteria were killed by washing twice in medium containing 200 μg/ml gentamicin (Invitrogen), followed by a further 1 hour incubation with medium containing gentamicin at this concentration. To continually exclude all extracellular bacteria, cells were subsequently maintained in medium containing 20 μg/ml gentamicin. At appropriate time points (2 or 24 hours), cells were washed twice with antibiotic-free medium and then lysed with PBS/0.1% Triton X-100. Lysates were cultured on LB agar plates at 37 °C and the number of colony forming units (CFU) was enumerated to assess intracellular bacterial loads. To assess bacterial uptake, macrophages were infected with bacteria for 1 hour. Cells were then washed twice with 200 μg/ml gentamicin, incubated in gentamicin-containing medium for a further 10 min, lysed, and the number of CFU was enumerated as described above. For both standard infection assays and uptake assays, complete exclusion of viable extracellular bacteria was confirmed by performing colony counts on culture supernatants.

### Semi-solid agar assay for assessment of motility

To evaluate motility, 5 μl of an overnight culture prepared in LB broth was spotted onto the centre or the edge of a freshly prepared 0.25% LB Bacto-agar plate (*n* = 4), supplemented with the appropriate inducer and/or antibiotic. Plates were incubated at 37 °C and the rate of motility was determined by measuring the diameter of the motility zone over time.

### Transmission electron microscopy (TEM)

Bacterial cultures were grown in LB broth at 37 °C overnight. Cells were washed with PBS and fixed in 2.5% (v/v) glutaraldehyde. A glow-discharged Formvar-coated copper grid was placed on a drop of the fixed cells suspension for 1 min to allow the cells to attach. Grids were washed in PBS and stained with 0.5% (w/v) uranyl acetate for visualization on a JEOL JEM 1010 TEM operated at 80 kV.

### Quantification of IL-10

To investigate the induction of IL-10 by isogenic flagella strains, co-cultures of 5637 uroepithelial cells and U937 monocytes were used. Methods for co-cultures and infections were as previously described^33^,^39^. The assays were performed in 96 well tissue culture plates using 50 μl aliquots of all cells in total volumes of 250 μl with RPMI medium. The bacteria were harvested from 0.25% LB agar cultures to enhance flagella expression. Incubation was for 5 hours at 37 °C with 5% CO_2_. Plates were centrifuged at 500 × *g* to pellet cells and supernatants were frozen at −80 °C until assay using an IL-10 ELISA (detection limit 2 pg/ml IL-10; eBioscience). Biological triplicates were performed and experiments were repeated at least three times. Data are pooled data from three independent experiments.

### Protein preparation and immunoblotting

Whole cells lysates were prepared by pelleting 1 ml of an overnight culture diluted to an optical density at 600nm (OD_600_) of 1.0, and resuspending in 50 μl of distilled water plus 50 μl of 2x SDS loading buffer. SDS PAGE and transfer of proteins to a PVDF membrane for western blot were performed as previously described^71^. Monospecific antisera against H1, H4 and H7 flagellin was purchased from the Statens Serum Institute, Denmark. Polyclonal antibodies against FimA (targeting the peptide CAGSVDQTVQLGQVRT) and LafA were generated by the Antibody Facility at the Walter and Eliza Hall Institute of Medical Research (Melbourne, Australia). OmpA antiserum was purchased from the Antibody Research Corporation, USA (item #111120). Primary antibodies were detected with commercially purchased alkaline phosphatase-conjugated anti-rabbit antibody (Sigma Aldrich). SIGMAFAST^TM^BCIP®/NBT (Sigma Aldrich) were used as substrate for detection.

### Statistical analysis

Data were analysed using either ANOVA, followed by a Tukey’s or Dunnett’s *post hoc* test to correct for multiple comparisons, for differences between more than two groups; or Student’s *t* test for differences between two groups. All analyses were performed using GraphPad Prism 6.0 (GraphPad Software, Inc.). *P* values of ≤0.05 were considered to be statistically significant.

## Additional Information

**How to cite this article**: Kakkanat, A. *et al.* The role of H4 flagella in *Escherichia coli* ST131 virulence. *Sci. Rep.* **5**, 16149; doi: 10.1038/srep16149 (2015).

## Supplementary Material

Supplementary Information

## Figures and Tables

**Table 1:** Bacterial strains and plasmids used in this study.

**Strains**	**Relevant characteristics**	**Reference**
EC958	ST131 reference strain; H4	36
CFT073	UPEC reference strain; H1	19
UTI89	UPEC reference strain; H7	18
EC958*fliC*	EC958*fliC*::cam; Cam^r^	This study
EC958*lafA*	EC958*lafA*::cam; Cam^r^	This study
EC958*fliClafA*	EC958*fliClafA*::cam; Cam^r^	This study
EC958^H1^	EC958*fliC*::*fliC*^H1^	This study
EC958^H4^	EC958*fliC*::*fliC*^H4^	This study
EC958^H7^	EC958*fliC*::*fliC*^H7^	This study
EC958*fimD*		36
EC958*fimDfliC*	EC958*fimDfliC*::cam;Cam^r^	This study
Plasmids		
p**F**lhDC	pMG600 derivative with Cam^r^ gene cassette from pKD3 inserted at EcoR1 site	This study
pMG600	pVLT33 derivative with *flhDC* genes	72
pVLT33	Broad-host-range expression vector	73
pVLT33a	pVLT33 with Cam^r^ gene cassette from pKD3 inserted at BamH1 site	This study
pKOBEG-**g**ent	λ-Red plasmid, Gent^r^	36,74
pCP20-**g**ent	FLP-recombinase plasmid, Gent^r^	66
pKD3	Template plasmid for Cam^r^ gene amplification	65
