# Supplementary material for: The role of H4 flagella in Escherichia coli ST131 virulence
Source: Sci Rep. 2015 Nov 9;5:16149. doi: 10.1038/srep16149 (PMC4637896; doi:10.1038/srep16149)
Supplement: Supplementary Information [file srep16149-s1.pdf]

Supplementary Information

The role of H4 flagella in *Escherichia coli* ST131 virulence

Asha Kakkanat[1], Makrina Totsika[2], Kolja Schaale[3], Benjamin L. Duell[4], Alvin W. Lo[1], Minh-Duy Phan[1], Danilo G. Moriel[1,2], Scott A. Beatson[1], Matthew J. Sweet[3], Glen C. Ulett[4*] and Mark A. Schembri[1*]

[1]*Australian Infectious Disease Research Centre, School of Chemistry and Molecular Biosciences, University of Queensland, Brisbane, Queensland, Australia.*

[2]*Institute of Health and Biomedical Innovation, School of Biomedical Sciences, Queensland University of Technology, Brisbane, Queensland, Australia*

[3]*Institute for Molecular Bioscience, University of Queensland, Brisbane, Queensland, Australia*

[4]*School of Medical Science, and Menzies Health Institute of Queensland, Griffith University, Gold Coast, Australia*

*** Corresponding authors:** m.schembri@uq.edu.au; g.ulett@griffith.edu.au.

Supplementary Information

Supplementary Figures S1-S9

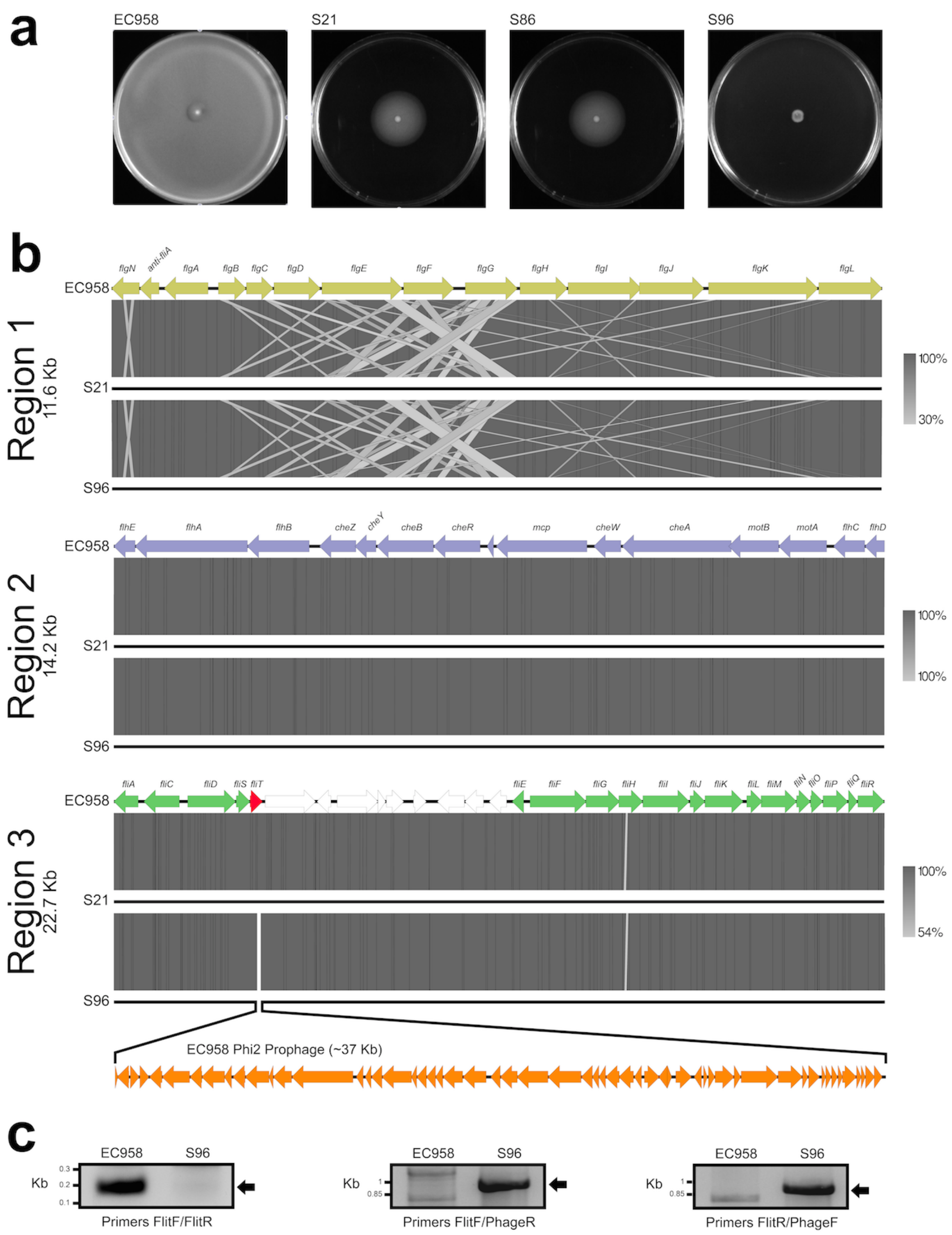

Fig. S1 Comparative genome analysis of flagellar regions of EC958 (motile), S21, S86 (strains displaying delayed motility) and S96 (non-motile strain). (a). Swimming phenotype of EC958, S21, S86 and S96 after 24 hours of incubation at 37°C. (b). Bioinformatic analysis of the three flagellar regions of EC958 compared to the corresponding region from S21 and S96 (prepared using Easyfig). Region 1 (yellow) and 2 (purple) are highly conserved among the three strains, while Region 3 (green) contains an insertion of the Phi2 prophage in *fliT* (red). In EC958, the Phi2 prophage (~37 kb) is present at a different genomic location. Genes depicted in white are not associated with flagella biosynthesis according to the literature. The regions are not drawn to scale. (c) PCRs to confirm the disruption of *fliT* by Phi2 prophage in S96, using *fliT* and Phi2 specific primers, and EC958 as a control.

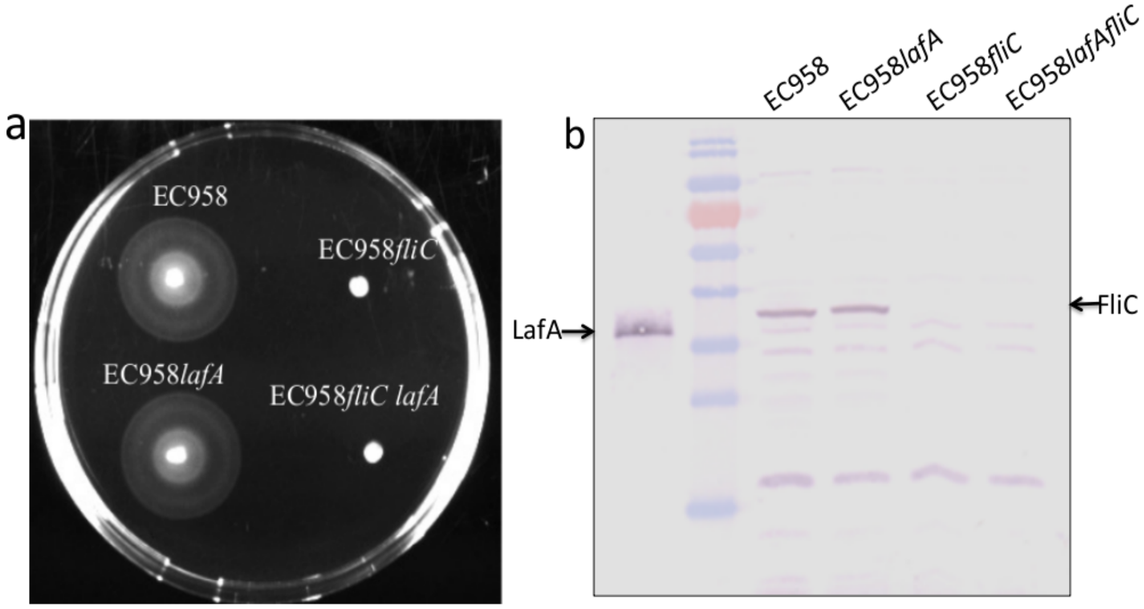

Fig. S2. The Flag-2 system of EC958 is non functional. (a). Motility of WT EC958, as well as EC958*fliC*, EC958*lafA* and EC958*fliC lafA* mutants in 0.25% LB agar. A volume of 5 µL of each bacterial strain grown in LB broth under shaking conditions was used as an inoculum. Shown is the motility of each strain after 6 hours of incubation at 37°C. (b). Western blot analysis using a LafA antibody in combination with whole cell lysates prepared from same strains cultured under shaking conditions at 37°C in LB broth. Lane 1, purified LafA protein (positive control), Lane 2, molecular weight markers, additional lanes represent strains as indicated. The LafA antibody was observed to cross-react with FliC; no cross-reacting band was detected in the two *fliC* mutant strains.

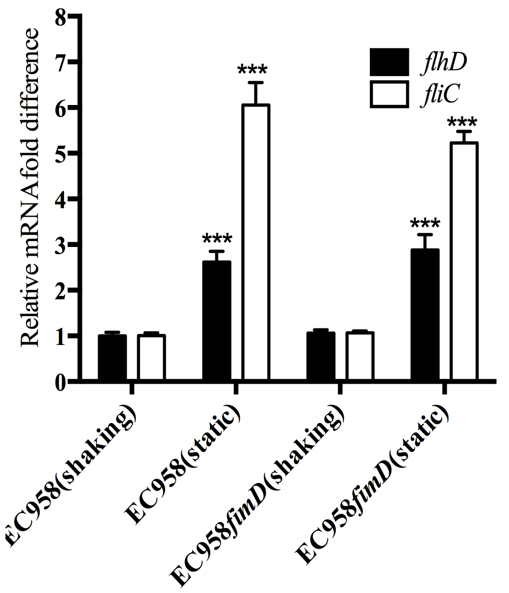

Fig. S3. Enhanced flagella expression by static growth is independent of type-1 fimbriae. Relative fold-difference of *flhD and fliC* transcript levels for EC958 and EC958*fimD* during growth in shaking and static conditions, determined by qRT-PCR. The data was obtained from three independent experiments; error bars indicate standard deviation. (***p<0.001)

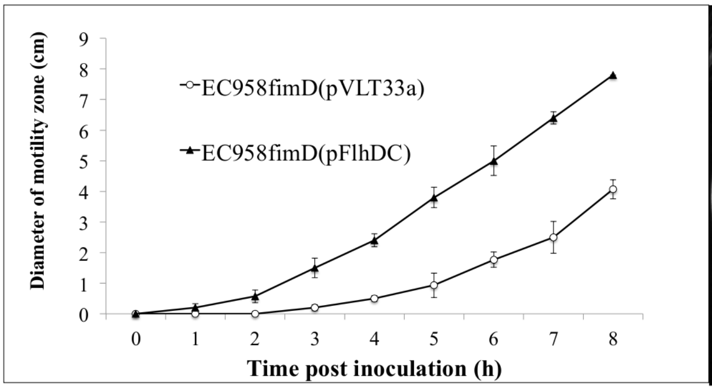

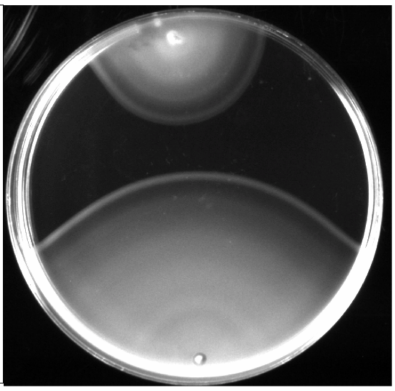

Fig. S4. FlhDC over-expression (via plasmid pFlhDC) leads to enhanced motility in EC958*fimD*. Graph demonstrating the rate of motility of EC958*fimD*(pVLT33a) and EC958*fimD*(pFlhDC) in 0.25% LB agar. Strains were grown overnight with 1mM IPTG induction in shaking LB broth cultures (left). Comparative motility of the same EC958 cultures in 0.25% agar at 6 hours post inoculation (right).

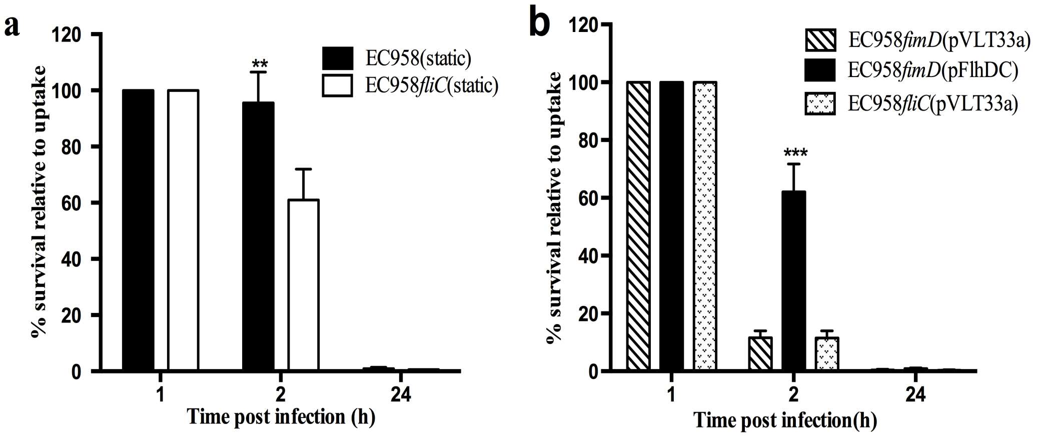

Fig. S5. Relative intramacrophage survival of (a) EC958 and EC958*fliC* grown under static conditions and (b) EC958*fimD*(pVLT33a), EC958*fimD*(pFlhDC) and EC958*fliC*(pVLT33a) grown under shaking condition Triplicate monolayers of BMM were infected at a MOI of 10. Intracellular bacterial loads were determined at 1, 2 and 24 hpi. Shown is the percentage bacterial survival relative to 100% uptake from three independent experiments ± standard deviation. (*** $p<0.001$; ** $p<0.01$)

```
                      20                    40
                       |                     |
H4 MAQVINTNSL SLITQNNINK NQSALSTSIE RLSSGLRINS AKDDAAGQAI 50
H1 .......... .......... ......S... .......... .......... 50
H7 .......... .......... ......S... .......... .......... 50
           60                    80                   100
            |                     |                     |
H4 ANRFTSNIKG LTQAARNAND GISLAQTAEG ALSEINNNLQ RIRELTVQAS 100
H1 .......... .......... ...V...T.. .......... .......... 100
H7 .......... .......... ...V...T.. .......... .......... 100
                     120                   140
                       |                     |
H4 TGTNSDSDLS SIQDEIKSRL DEIDRVSGQT QFNGVNVLSK NDSMKIQIGA 150
H1 .........D .......... .......... ........A. DG.....V.. 150
H7 .........D .......... .......... ........A. DG.....V.. 150
          160                   180                   200
            |                     |                     |
H4 NDNQTISIGL QQIDSTTLNL KGFTVSG--- -------MAD FSAAKLTAAD 190
H1 ..G...T.D. KK...D..G. N..N.N.SGT IANKAATIS. LT...MD..T 200
H7 ..GE..T.D. KK...D..G. N..N.N.KGT ITNKAATVS. LTS.GAKLNT 200
                     220                   240
                       |                     |
H4 GT-------- --AIAA---- --------AD VK-------- ---------- 200
H1 N.----ITTT NN.LT.SKAL DQLKDGDTVT I.----ADAA QTATVYTYNA 242
H7 T.GLYDLKTE NTLLTTDAAF DKLGNGDKVT .GGVDYTYNA KSGDFTTTKS 250
          260                   280                   300
            |                     |                     |
H4 ---------- ---------- ---------- ---------- ---------- 200
H1 SAGN-FSFSN VSNNTSAKAG DVAASLLPPA GQTASGVYKA ASGEVNFDVD 291
H7 TAGTGVDAAA QATDSAKKRD ALAATLHADV GKSVNGSYTT KDGTVSFETD 300
                     320                   340
                       |                     |
H4 ---------- ---------- ---------- ---------- ---------- 200
H1 ANGKITIGGQ EAYLTSDGNL TTNDAGGATA ATLDGLFKKA GDGQSIGFNK 341
H7 SAGNITIGGS QAYVDDAGNL TTNNAGSAAK ADMKALLKAA SEG-----SD 345
          360                   380                   400
            |                     |                     |
H4 ---------- -------DAG GKQVN----- LLSYTDTASN STKYAVVDSA 228
H1 TASVTMGGTT YNFKTGA... AATA.----A GV.F.....K E.VLNK.AT. 387
H7 GASLTFNGTE YTIAKATP.T TSP.APLIPG GIT.QA.V.K DVVLSETKA. 395
                     420                   440
                       |                     |
H4 TG-------- ---------- ---------- ---------- ---------- 230
H1 KQGTAVAANG DTSATITYKS GVQTYQAVFA AGD------- ------GTAS 424
H7 AA-------- --TSSITFNS GVLSKTIGFT AGE------- -----SSDAA 423
          460                   480                   500
            |                     |                     |
H4 -KYMEATVVI TGTA------ ---------- ---------A AVTVG----- 249
H1 A..ADN.D.S NA..TYTDAD GEMTTIGSYT TKYSIDANNG K...D---SG 471
H7 KS.VDDKGG. .NV.DYTVS- ---------- --YSVNKDNG S...AGYASA 460
                     520                   540
                       |                     |
H4 ---------- ---------- -----AAEVA GAATADPLKA LDAAIAKVDK 274
H1 TGTGK-YAPK VGAEVYVSAN GTLTTD.TSE .TV.K..... ..E..SSI.. 520
H7 TDTNKDYAPA IGTAVNVNSA GKITTETTS. .S..TN..A. ..D..SSI.. 510
          560                   580                   600
            |                     |                     |
H4 FRSSLGAVQN RLDSAVTNLN NTTTNLSEAQ SRIQDADYAT EVSNMSKAQI 324
H1 .......I.. .......... .......... .......... .......... 570
H7 .......I.. .......... .......... .......... .......... 560
                     620
                       |
H4 IQQAGNSVLS KANQVPQQVL SLLQG 349
H1 .........A .......... ..... 595
H7 .........A .......... ..... 585
```

Fig. S6. Amino acid alignment of the H4 (EC958), H1 (CFT073) and H7 (UTI89) flagellins. Sequence alignments were performed using CLC main workbench 7.0.2. Residues identical to those of the H4 flagellin are indicated by dots.

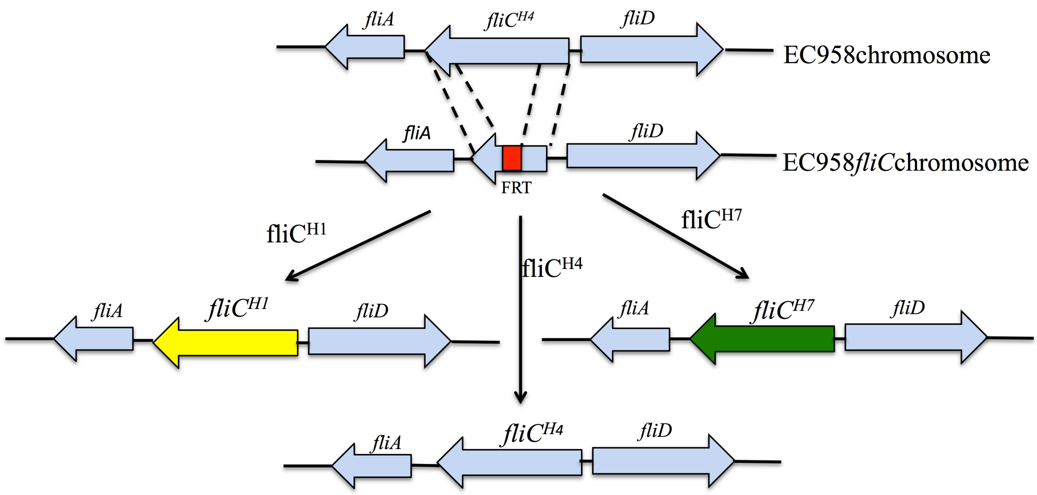

Fig. S7. Schematic representation of the method used for markerless replacement of the *fliC* allele in the EC958 chromosome. In the first step, the *fliC* gene was mutated by insertion of a chloramphenicol gene cassette using λ-Red recombination. The chloramphenicol cassette was subsequently removed by the introduction of plasmid pCP20, resulting in a large deletion in *fliC*. Next, the *fliC* gene from CFT073 (H1) and UTI89 (H7) and EC958 (H4; control) was amplified with 500bp homologous arms matching the EC958 *fliC* sequence; colour-coded as yellow, green and blue. These cassettes were then transformed into EC958*fliC* by electroporation and the transformation mix was spotted onto the centre of a 0.25% LB agar plate. Isogenic strains containing a reconstructed *fliC* allele were selected by their ability to swim through the agar.

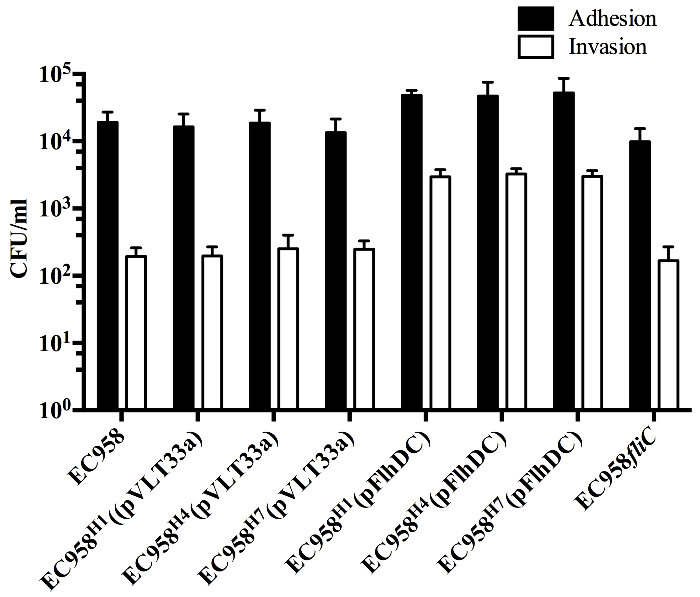

Fig. S8. Isogenic EC958 flagella variants show similar level of adhesion and invasion. Adhesion to and invasion of T24 bladder epithelial cells by EC958, EC958*fliC* (controls), EC958^{H1} (+/- pFlhDC) EC958^{H7} (+/- pFlhDC) and EC958^{H4} (+/- pFlhDC). Bacterial strains were grown under shaking conditions. Monolayers were infected in triplicate, and data represents the mean CFU/ml from three independent experiments ± standard deviation.

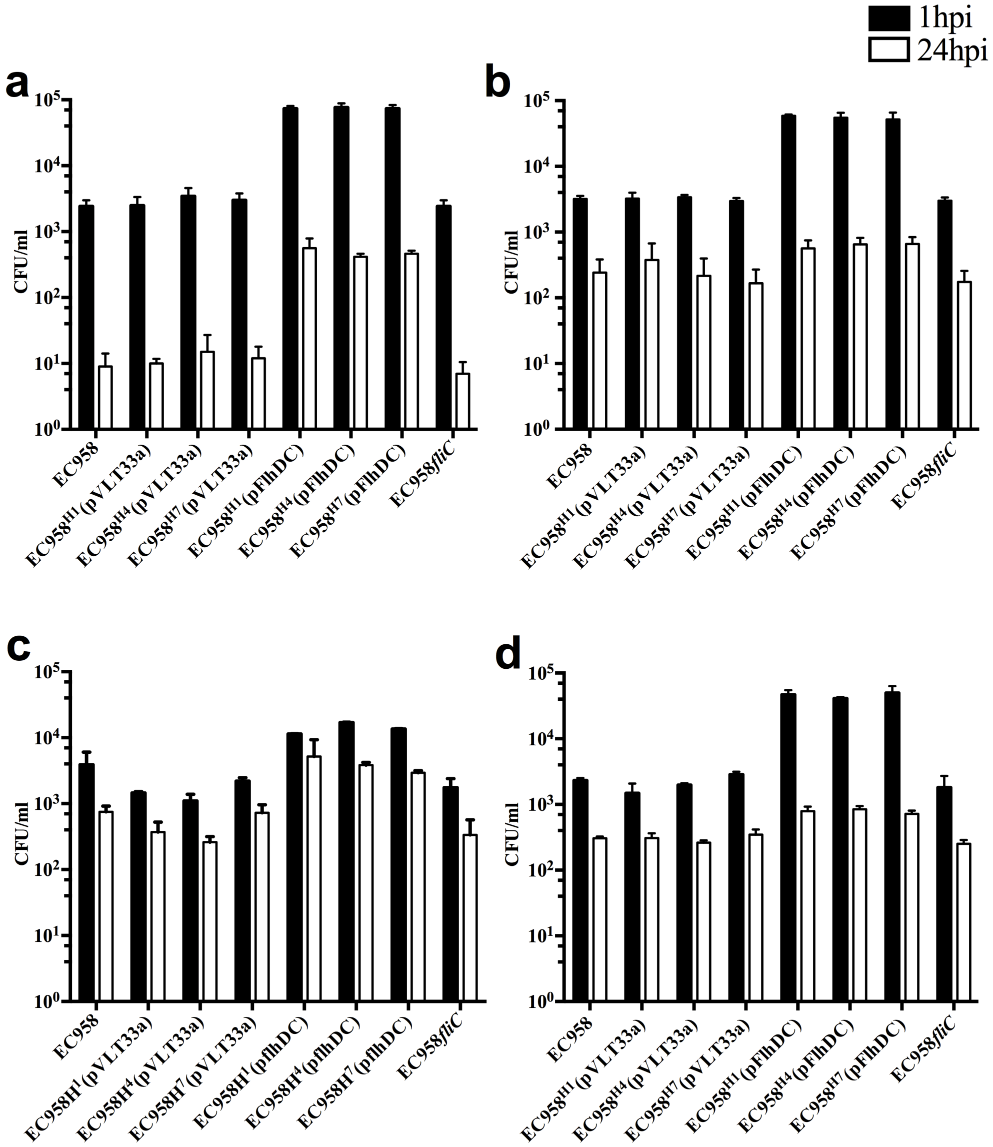

Fig. S9. Isogenic EC958 flagella variants show similar intramacrophage survival. Intracellular survival of EC958, EC958*fliC* (controls), EC958^{H1} (+/- pFlhDC) EC958^{H7} (+/- pFlhDC) and EC958^{H4} (+/- pFlhDC) in (a) BMM (b) HMDM (c) THP-1 cells and (d) U937 cells. Bacterial strains were grown under shaking conditions. Cells were infected at an MOI of 10 and intracellular survival was measured at 1 (black bar) and 24 (white bar) hpi. Shown is the mean CFU/ml from three independent experiments ± standard deviation.
